# Development and Application of Novel Sodium Silicate Microcapsule-Based Self-Healing Oil Well Cement

**DOI:** 10.3390/ma13020456

**Published:** 2020-01-17

**Authors:** Wenting Mao, Chrysoula Litina, Abir Al-Tabbaa

**Affiliations:** 1 Yunnan Institute of Building Research, 150 Xuefu Road, Kunming 650223, China; wm280@alumni.cam.ac.uk; 2 Department of Engineering, University of Cambridge, Trumpington Street, Cambridge CB2 1PZ, UK; cl519@cam.ac.uk

**Keywords:** oil well cement, high temperature, self-healing, sodium silicate microcapsules

## Abstract

A majority of well integrity problems originate from cracks of oil well cement. To address the crack issues, bespoke sodium silicate microcapsules were used in this study for introducing autonomous crack healing ability to oil well cement under high-temperature service conditions at 80 °C. Two types of sodium silicate microcapsule, which differed in their polyurea shell properties, were first evaluated on their suitability for use under the high temperature of 80 °C in the wellbore. Both types of microcapsules showed good thermal stability and survivability during mixing. The microcapsules with a more rigid shell were chosen over microcapsule with a more rubbery shell for further tests on the self-healing efficiency since the former had much less negative effect on the oil well cement strength. It was found that oil well cement itself showed very little healing capability when cured at 80 °C, but the addition of the microcapsules significantly promoted its self-healing performance. After healing for 7 days at 80 °C, the microcapsule-containing cement pastes achieved crack depth reduction up to ~58%, sorptivity coefficient reduction up to ~76%, and flexural strength regain up to ~27%. The microstructure analysis further confirmed the stability of microcapsules and their self-healing reactions upon cracking in the high temperature oil well cement system. These results provide a promising perspective for the development of self-healing microcapsule-based oil well cements.

## 1. Introduction

As the dominant construction material, cement has been widely used in construction applications worldwide, not only for civil infrastructures but also for a range of more tailored applications including wellbores in oil fields. Cementing is one of the most important and critical operations performed on oil and gas wells [1]. It is the process of pumping down cement slurry to form a cement sheath and hydraulically sealing the annulus between the casing and the rock formation. The formed cement sheath delivers zonal isolation, completely sealing off oil and gas from the wellbore, preventing fluid communication between producing zones in the borehole, and blocking the escape of fluids to the surface. The cement sheath hence becomes subject to external and internal stresses, including thermal stresses from cement hydration or injection of steam/cold fluids, shrinkage-induced stresses and mechanical stresses caused by the drill pipe and other tubulars banging in the casing, which begin at the time a wellbore is cemented and throughout the lifetime of the wellbore [2]. 

For decades, the service industry has worked on improving the formulation, placement and properties of hardened cement. Downhole cement systems need to be designed to perform over a wide temperature range, from below freezing in permafrost zones to temperatures exceeding 500 °C in geothermal wells, and pressure range from near ambient in shallow wells to more than 200 MPa in deep wells [1]. Conventional oil well cement systems exhibit unfavorable characteristics that adversely affect their sealing capacity such as their brittle nature, shrinkage and weak bonding to the casing and formation. From a cementing perspective, high shrinkage leads to increased porosity and stresses in the cement sheath. The imposed stresses therefore have the potential to induce cracks in the cement column and create microannuli [2]. Weak bonding can cause failure at cement-casing or cement-formation interfaces [3,4]. These defects in the cement sheath could adversely affect their sealing capacity to provide zonal isolation, resulting in gas/liquid migration through the flow paths created by cement failure and even serious leak or blowout accidents. Poor cementing has been linked to the Macondo oil rig disaster in the Gulf of Mexico in 2010 [5] and in the 2011 SPE Forum on ‘Drilling and Completing Trouble Zones’, it was reported that 45% out of 15,000 active wells in the Gulf of Mexico have sustained casing pressure (Shell, Personal communication). In addition, the cost of well abandonment at the end of the productive time of an oil or gas well will significantly increase due to poor zonal isolation. A further challenge is that the industry has been pushing for tighter casing schemes, including the use of Expandable Casing, and these developments will lead to narrower annular gaps making cement placement and hardening without cracks even more difficult.

The conventional solution to the cracking problems is remedial cementing through the injection of cement slurry into cracking locations, which is usually untimely and ineffective [1]. This is also an extremely costly process and the inaccessible nature of the wellbore means that it is very difficult to inspect the damage and repair processes. This has fueled interest in the development of self-healing oil well cements that would have an in-built capability to repair micron-sized cracks autonomously without any external intervention.

Portland cement has a limited inherent capability to self-heal small-width cracks in the appropriate curing environment, mainly curing under water, as a result of the further hydration of unhydrated cement particles and the formation of calcite (CaCO_3_) through carbonation, and this process is referred as autogenous healing [6]. In the extreme downhole environment, autogenous healing would be expected to be extremely limited. The elevated operating temperatures will significantly accelerate and enhance the cement hydration process resulting in very little residual unhydrated cement being available for autogenous healing. In addition, water is not freely available at rock formation level. Hence any inherent autogenous healing capability cannot be relied upon to seal any crack sizes.

**Figure 1:**
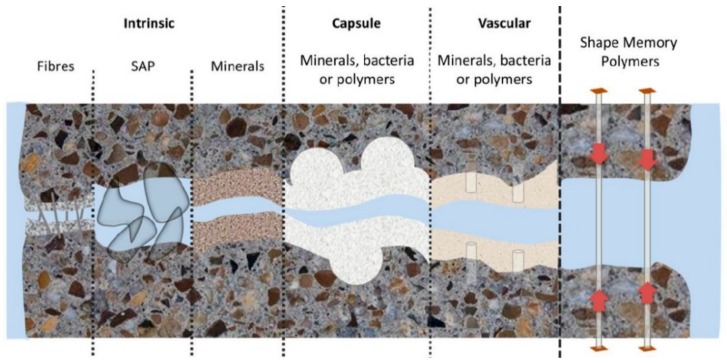
Autonomic self-healing systems developed for cementitious materials, depending on the crack size [7].

The self-healing ability of cementitious systems can be enhanced by engineered additions of external healing agents, and this usually depends on the crack size and the nature of the cementitious matrix [6] as shown in Figure 1 [7]. Enhanced autogenous healing can be facilitated through non-encapsulated additions which include fibers, mineral or crystalline additives and superabsorbent polymers. Reactive magnesia has been researched as an expansive additive for this application to provide prestressing to increase the threshold for tensile cracking [8]. Oil/hydrocarbon swellable polymers were also developed to modify the cement paste to improve the crack self-healing property triggered by oil/gas leakage. When oil/gas from the formation come into contact with the polymer particles, the particles swell and then block the crack [9,10,11].

For larger damage, microencapsulated healing agents embedded in conventional cementitious matrices have been proposed [12]. When a crack occurs, the pre-embedded microcapsules break and the healing agent is released to seal the cracks. The sealing mechanism and rate are highly dependent on the type and properties of the cargo used. Polymeric, biological and mineral cargos have been investigated in cementitious systems, with the latter being the most viable for the high temperature downhole environment and in general for compatibility and stability with cementitious systems [13].

Mineral healing agents rely on chemical reactions with the cement matrix or with water or CO_2_, producing hydration and carbonation products similar to those that take place within the cementitious matrix under its natural hydration and carbonation processes. Examples of suitable mineral additives include MgO, CaO and silica-based minerals which generally possess good stability under high temperature as well as high pressure conditions as encountered by oil well cement systems. Amongst the mineral-based additives, sodium silicate is the most frequently investigated agents for autonomic self-healing cementitious materials. It can react with calcium hydroxide, a main product of cement hydration, and produce calcium silicate hydrate (C-S-H) gel which is the natural binding material in cementitious matrices. The newly formed C-S-H gel acts as a binder and healer in cracks and pores, bridging the gaps between cracking surfaces [14,15,16,17,18].

Microcapsules containing sodium silicate cargo for self-healing applications in cementitious systems, is a recent development and has only been reported in a handful of studies [14,16,19,20,21,22]. Those microcapsules have been produced in different size ranges and with different shell materials and tested mainly for strength recovery in cementitious systems for conventional construction applications. The microcapsule shell wall, the interface between the cargo and the cementitious matrix, plays a vital role in ensuring the survival of the microcapsules during the mixing and pumping processes and its subsequent stability in the highly alkaline environment of the cementitious matrix. Among these materials polyureathen (PU) and polyurea (PUrea) properties and compatibility has been considered to meet the demanding criteria required for microencapsulated based self-healing systems. PU and PUrea shells allow the inclusion of aqueous compounds which has great potential in delivering inorganic salts and minerals [23,24,25,26]. The promising results in those studies, both in terms of the rupture of the microcapsules by crack propagation and the release of the sodium silicate and subsequent healing of cracks [27,28] provided the impetus to apply some of the same microcapsules for self-healing in oil well cement systems. In oil well cement applications, there is the added challenge of their survivability in the high temperature environment for which the thermal stability of the shell materials is of primary concern. The PUrea generally possesses good thermal stability up to ~230–240 °C [23,29], which makes these microcapsules suitable for application in such high temperature conditions. This paper presents details of the development of sodium silicate-based self-healing microcapsules for oil well cements under high temperature wellbore environment. Typically, the temperature from 50 °C to 120 °C covers 90% of the oil/gas wells and 80 °C was chosen as the target temperature in this study. Two types of sodium silicate microcapsules with different shells were investigated. They were first evaluated for their survivability under the high temperature wellbore conditions, by examining their thermal stability and resistance to alkalinity. The microcapsules were then evaluated for their effects on the rheological properties and hydration processes of fresh oil well cement slurry, as well as the compressive strength of hardened oil well cement pastes. Based on these evaluation results, the more suitable microcapsule type was selected for further investigation of their self-healing performance in oil well cement at 80 °C through water sorptivity tests. Microstructural analyses, including SEM and EDX, were carried out to observe the microcapsules in the cement matrix and investigate the healing products formed.

## 2. Experimental Program

### 2.1. Materials

**Figure 2:**
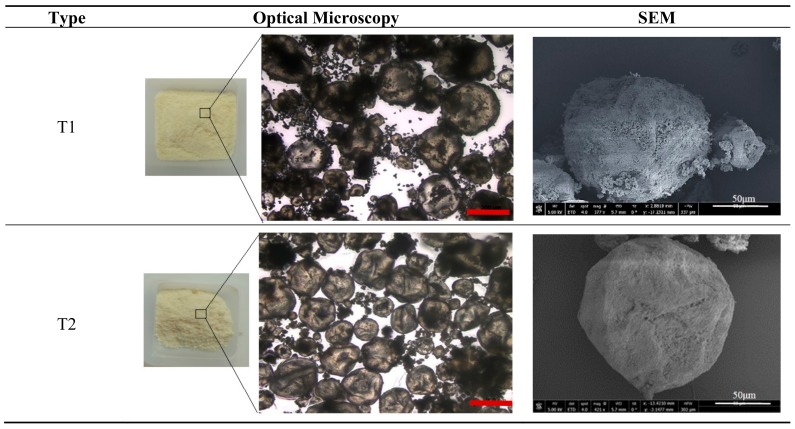
Optical and SEM images of the T1 and T2 microcapsules used in this study.

Two types of polymeric microcapsules, referred to here as T1 and T2, were used for the self-healing study in oil well cement. The microcapsules were produced by Thies Technology Inc (Henderson, NV, USA) using interfacial polymerization with polyurea as the shell wall and sodium silicate as the core material, a part of a joint collaboration between us, and were delivered in dry form. Due to the highly acidic conditions of the microcapsule production process, the encapsulated sodium silicate polycondenses to a semi-crystalline state within the shell. Both microcapsule types had the same average size of 110 μm with a slightly narrower range for T1 of 30–220 μm than T2 of 20–250 μm. The microcapsules generally consisted of ~90% sodium silicate core materials and ~10% polyurea shells by weight. The two mainly differ in their polyurea shell where the T1 microcapsules have a rigid shell and the T2 microcapsules have a rubbery shell. Typical optical and SEM images of the microcapsules are shown in Figure 2. The microcapsules present as globular shapes surrounded by some tiny debris which might be either from the shell material or unencapsulated sodium silicate.

The American Petroleum Institute (API) specifications Class G oil well cement, the most widely used in oil well cementing applications, was used for preparing the cement paste specimens. The cement was supplied by Dyckerhoff (Wiesbaden, Germany).

### 2.2. Thermal Stability and Chemical Resistance Analyses of the Microcapsules

Thermogravimetric analysis (TGA), conducted using a METTLER TOLEDOTGA/DSC 1 instrument (Columbus, OH, USA), was used for characterizing the thermal stability of the microcapsules, and their polyurea shell material. The shell material was extracted by grinding the microcapsules and dissolving the sodium silicate core using water to separate it from the polyurea shell. The suspension was then filtered to obtain the shell debris which was dried in an oven at 50 °C to remove the moisture. A small quantity, ~3–5 mg, of microcapsule samples and the shell materials was placed in a ceramic crucible and heated within the temperature range of 50 to 650 °C at a rate of 10 °C/min in a nitrogen environment. To examine the chemical stability of the microcapsules in combined high temperature and high alkaline conditions, the two microcapsule types were immersed in saturated Ca(OH)_2_ solution (pH ≈ 13) at a temperature of 80 °C that simulated the alkaline cementitious environment and the wellbore temperature. After exposure of 14 days, the microcapsules were observed using an optical microscope.

### 2.3. Preparation of Oil Well Cement Pastes and Samples

The oil well cement pastes were prepared at a constant water cement (*w*/*c*) ratio of 0.44. The microcapsules were added into the cement at different contents varying from 0 to 7.5% by weight of cement. The microcapsules were first mixed thoroughly with the dry cement powders for ~5 min, and then water was added and mixed according to the requirements of API specification [30]. After mixing, the cement paste was placed in cubic molds (40 mm × 40 mm × 40 mm) and prismatic molds (40 mm × 40 mm × 160 mm), and then compacted using a vibrating table. The prismatic specimens were cast with a steel wire (1.6 mm in diameter and 130 mm in length) placed in the compressive zone of the prism with a cover of 10 mm from the top to prevent complete sample disintegration under loading. The samples in the molds were then covered with multiple layers of plastic film to prevent loss of water through evaporation during the curing stage, and were then left to cure for 6 h in the incubator (relative humidity > 95%) at the specified temperature of 80 °C, to simulate both the moisture and temperature environment downhole. The 6 h allowed demolding and the hardened cement samples were further cured for 3 days in the same environment before testing.

### 2.4. Rheology, Compressive Strength and Water Sorptivity Tests

A Brookfield DV3T Rheometer (Harlow, Essex, UK) was used to assess the effect of the microcapsules on the rheological properties (plastic viscosity and yield stress) of the cement slurries. The *w/c* ratio was kept constant at 0.44 and the cement slurries were prepared by mixing 30 g cement, 13.2 g water and the corresponding microcapsules in varying quantities together in a plastic cup using a vortex mixer. After mixing, the cement slurries were placed into the rheometer sample cup for testing.

Compressive strength tests were performed on the cubic cement paste samples to evaluate the effect of microcapsules on the strength of oil well cement pastes. After curing for 3 days at 80 °C, the cubes were tested for their unconfined compressive strength (UCS) using a 250 kN servohydraulic testing frame with a Controls 50-C9030 compression device (Norwood, MA, USA) at the loading rate of 2400 N/s in accordance with BS EN 196-1 [31] and API Specification 10A [30]. The samples were tested in triplicate and the average value taken.

**Figure 3:**
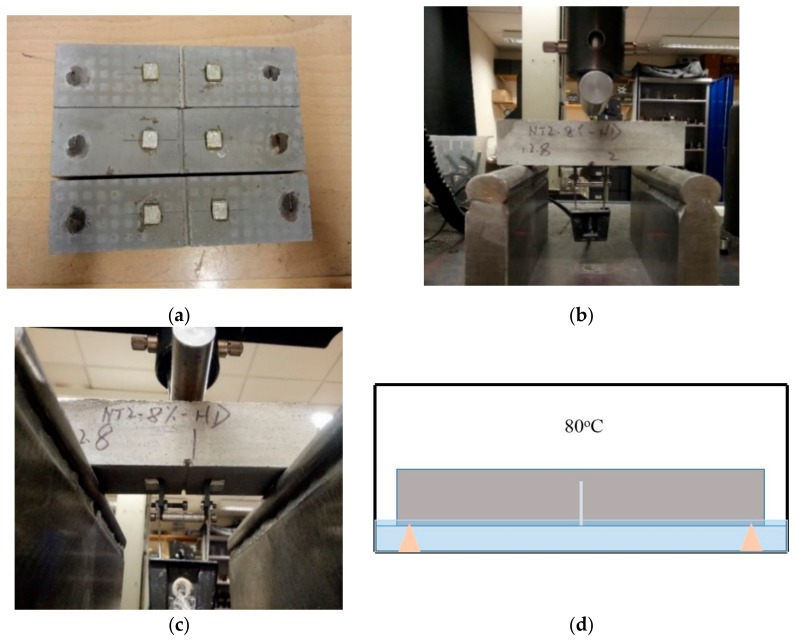
The sample preparations for the water sorptivity test (**a**) the notched 40 mm × 40 mm × 160 mm prisms with the two knife edges fixed on both sides of the notch, (**b**) the three-point bending test set-up, (**c**) the control mechanism of crack width using a clip gauge attached to the glued knife edges at the base of the specimens and (**d**) the curing environment used for the cracked prism specimens at 80 °C.

After 3 days of curing at 80 °C, the prismatic specimens were cracked under three-point bending for water sorptivity testing. Prior to cracking, the specimens were notched (1.5 mm depth and 2 mm width) at their base side in order to ensure the crack initiated in the center of the specimens (Figure 3a). The notched samples were then mechanically cracked using an Instron 30 kN static testing frame at a rate of 0.10 mm/min (Figure 3b). The crack width was controlled using a clip gauge that was attached to the base of the specimens through two knife edges fixed on both sides of the notch (Figure 3c). The cement prisms were loaded to a crack mouth opening displacement (CMOD) of 200 µm. The cracked specimens were then cured in the incubator for another 7 days at 80 °C in a sealed container containing water at a level of 2–3 mm above the base of the specimens, as shown in Figure 3d. The water was absorbed into the crack by capillary force to assist the healing process.

**Figure 4:**
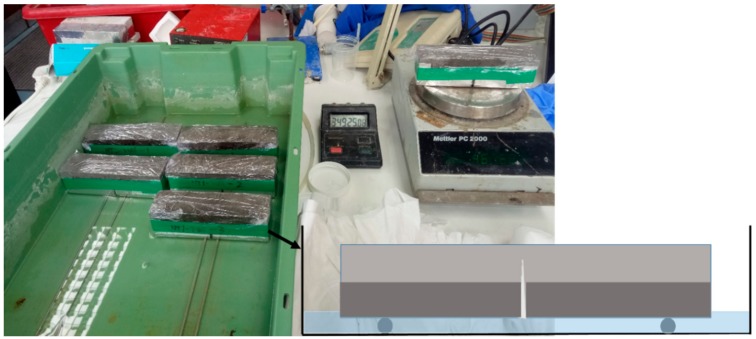
Illustration of the capillary water absorption testing procedure.

The water sorptivity test was carried out on the cracked prismatic specimens after 7 days of healing following the testing procedure in ASTM C1585-13 [32] and RILEM report [6], to evaluate their recovery in terms of their water tightness. As shown in Figure 4, the cracked prism specimens were first dried in a vacuum chamber until the mass changes were less than 0.1% between 24 h periods. The specimens were then wrapped around their base and sides with aluminium tape but the crack area was left uncovered for exposure to capillary water suction. The top side of the specimens was also covered with cling film to prevent moisture evaporation from the specimens during the test. Specimens were placed on supports in a tank containing a shallow water level which was maintained at ~3 mm throughout the test. Weight changes in the specimens due to capillary water uptake were monitored at the specified time intervals over ~4.5 h. The sorptivity coefficient was calculated based on Equations (1) and (2) [33]:

(1) $$M_{w}=\frac{\Delta m}{A\rho }$$

(2) $$M_{w}=C+S\sqrt{t}$$

where *M_w_* is the water uptake quantity per unit area of inflow surface (mm), Δm is the change in specimen weight (g), A is the area of inflow surface (mm^2^), ρ is water density (g/mm^3^), and *S* is the sorptivity coefficient (mm/min^1/2^) determined by calculating the slope of curve between *M_w_* and the square root of measurement time t (min^1/2^).

### 2.5. Ultrasonic Pulse Velocity Test on Crack Depth

**Figure 5:**
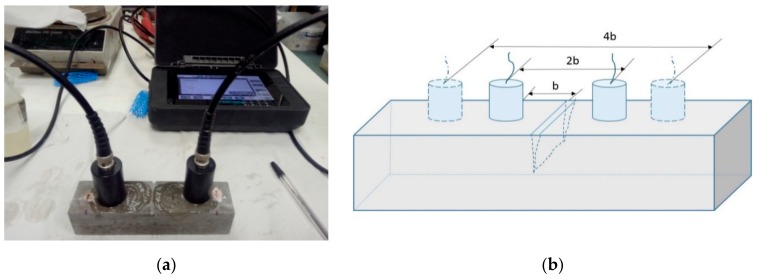
The ultrasonic velocity test used for crack depth measurement (**a**) the instrument PUNDIT-PL 200 used and (**b**) a schematic diagram of the crack depth measurement principle.

A non-destructive technique using ultrasonic pulse velocity test equipment was employed to measure the depth of the cracks in the oilwell cement prisms after healing for 7 days at 80 °C. This method is based on the fact that ultrasonic waves travel much easier in hardened cementitious matrix (4000–5000 m/s) than in water (1480 m/s) or air (350 m/s). As a result, they will travel around the open cracks leading to an increase in transmission time. When the crack is sealed, the waves will be able to travel through the formed healing products that fill the cracks, thus reducing the transmission time. By measuring the wave transmission time, the depth of cracks can be calculated. As illustrated in Figure 5, two measurements were taken of the ultrasonic wave transmission time of the pulse to transit distances 2b and 4b, respectively. The time (*t*_1_ and *t*_2_) taken for the ultrasonic waves to travel between the two points was recorded and the crack depth d was then calculated based on Equation (3).

(3) $$d=b\sqrt{\frac{4t_{1}^{2}-t_{2}^{2}}{t_{2}^{2}-t_{1}^{2}}}$$

### 2.6. Microstructural Analysis

Scanning electron microscopy (SEM) and energy dispersive X-ray spectroscopy (EDX) were used to verify the self-healing reaction between the sodium silicate core material and the cement. Small chips were collected from the crack surface of prism samples containing microcapsules and tested using a FEI Nova NanoSEM FEG machine (Hillsboro, Oregon, USA).

## 3. Results and Discussion

### 3.1. Thermal Stability and Chemical Resistance of the Microcapsules

**Figure 6:**
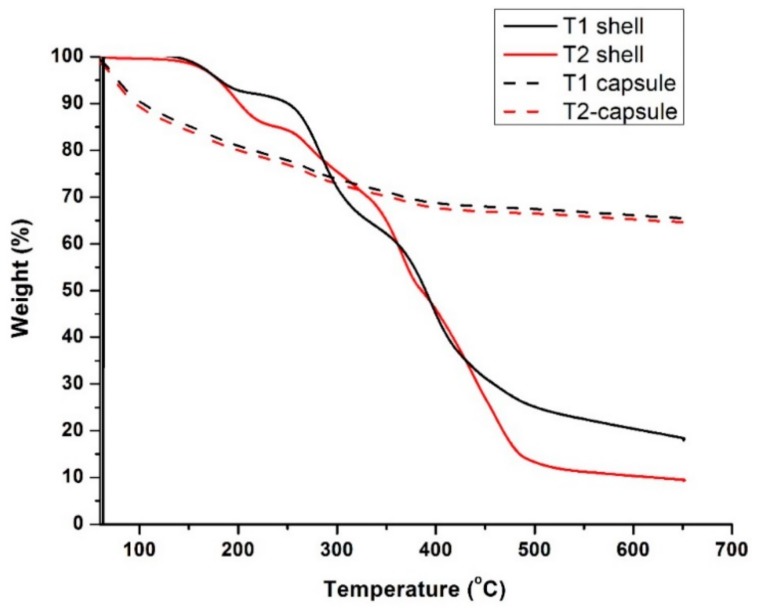
TGA analysis on two types of microcapsules and their shell materials.

Figure 6 presents the thermogravimetric traces of the T1 and T2 microcapsules together with their shell materials. Both sets of traces displayed similar trends that showed the first weight depletion from the initial temperature of 50 °C and experienced a gradual weight loss over the following wide temperature range up to ~400 °C, reaching a residual weight of ~70% at 650 °C. On the other hand, the shell material alone for both microcapsules generally remained thermally constant up to ~160 °C and then decomposed gradually from ~150 to ~490 °C, finally reaching a residual weight of ~19% for T1 and ~10% for T2 at 650 °C. Similar trends in the thermogravimetric traces of polyurea-type microcapsule shells were reported in the literature [25,34]. By comparing the thermal behavior of the capsules and the shell materials, it can be deduced that the initial weight loss of the capsules from 50 °C can be mainly attributed to the water loss from the hydrous sodium silicate core. Sodium silicate hydrates can start dehydrating at the low temperature of ~40 °C and transform into metasilicate above ~240 °C [35]. The microcapsules are deemed to remain intact up to the initial decomposition temperature of the shell wall (T1 at 150 °C and T2 at 160 °C), which are far above the target temperature of 80 °C.

**Figure 7:**
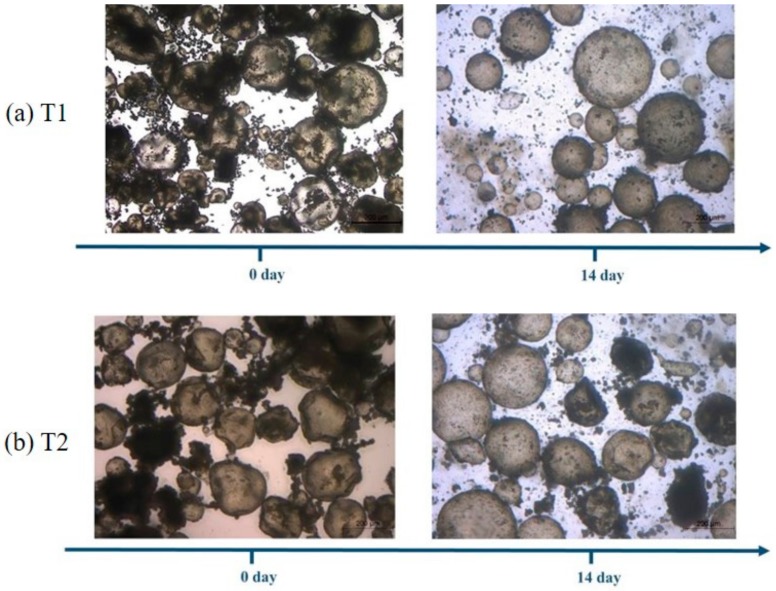
Stability of the two types of polyurea/sodium silicate microcapsules in saturated Ca(OH)_2_ solution (pH ≈ 13) at 80 °C at 0 and at 14 days (**a**) the T1 microcapsules and (**b**) the T2 microcapsules.

After an exposure of 14 days in saturated Ca(OH)_2_ solution (pH ≈ 13) at 80 °C, both types of microcapsules were able to retain their shell integrity, as shown in Figure 7, and hence the shell wall acted as an important protection layer for the cargo. The polyurea shells had good thermal stability as demonstrated by the thermogravimetric results above. Polyurea materials are also reported to have good resistance to alkalis [36], which enabled the microcapsules to survive in the high temperature and high alkaline condition. Hence, it can be concluded that both T1 and T2 microcapsules are suitable for use under the high temperature and high alkaline condition of oil well cement systems.

### 3.2. Effect of the Microcapsules on the Rheological Properties of the Oil Well Cement Slurries

**Figure 8:**
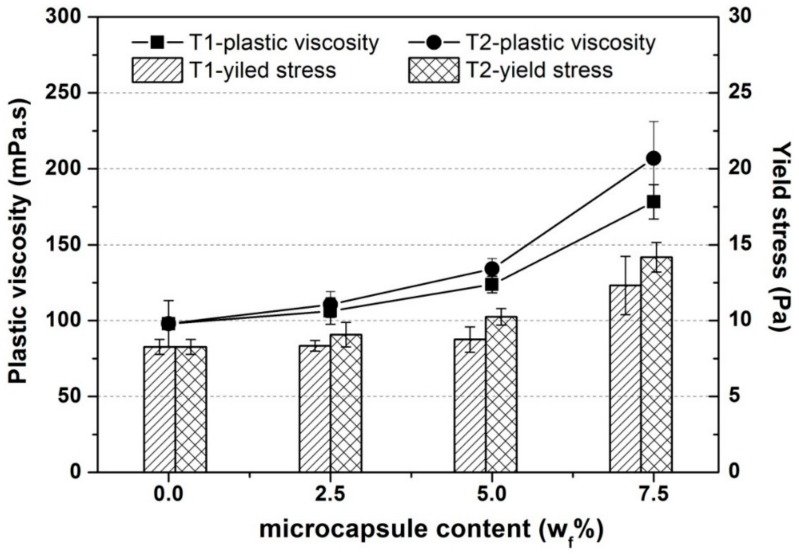
Plastic viscosity and yield stress of the oil well cement slurries containing the polyurea/sodium silicate microcapsules at varying dosages of the T1 and the T2 microcapsules.

Figure 8 presents the variation in the plastic viscosity and yield stress of the oil well cement slurries with increasing additions of the T1 or T2 microcapsule from 0 to 7.5% at 20 °C. In general, T1 and T2 microcapsules showed similar influencing trends on the rheological properties. Increasing the dosages from 0 to 5%, led to a linear increase of the plastic viscosity and yield stress while a much more significant increase was observed with the higher dosage content of 7.5%. Similar trend was also reported by other researchers [16,22] in the same group who used spherical polymeric microcapsules. For a dilute phase, where the concentration of particles is low, the relative motion of fluid near and around them remains largely unaffected by the volume fraction of particles [22]. For such dilute phases, Einstein had found that their viscosity is correlated linearly with increasing particle concentration. As the volume fraction of microcapsules increases, it induces the so-called crowding effect [37,38,39]. The frictional contact force between the particles also increases and obstructs the movement of fluid around the particles, resulting in increased viscosity. The large particles with interfering sizes are crowded mutually and the small particles decreases the free space for large particles. The small particles filling the spaces between the large particles to reduce the free spaces which is usually larger than the particle size. Particles with different effective volumes when crowded can result in dead fluid trapped between them resulting in rapid decrease of the flowability of the suspension [40]. This explains the significant influence of the microcapsules on the yield stress for the 7.5% dosage. As the flowability decreased, a higher energy level was needed to initiate flow of the cement slurries, thus increasing the yield stress.

### 3.3. Survivability of the Microcapsules in the Cement Pastes

**Figure 9:**
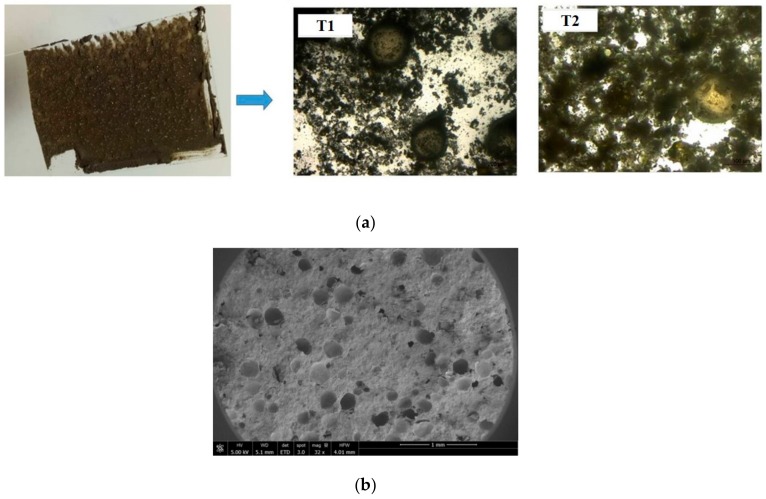

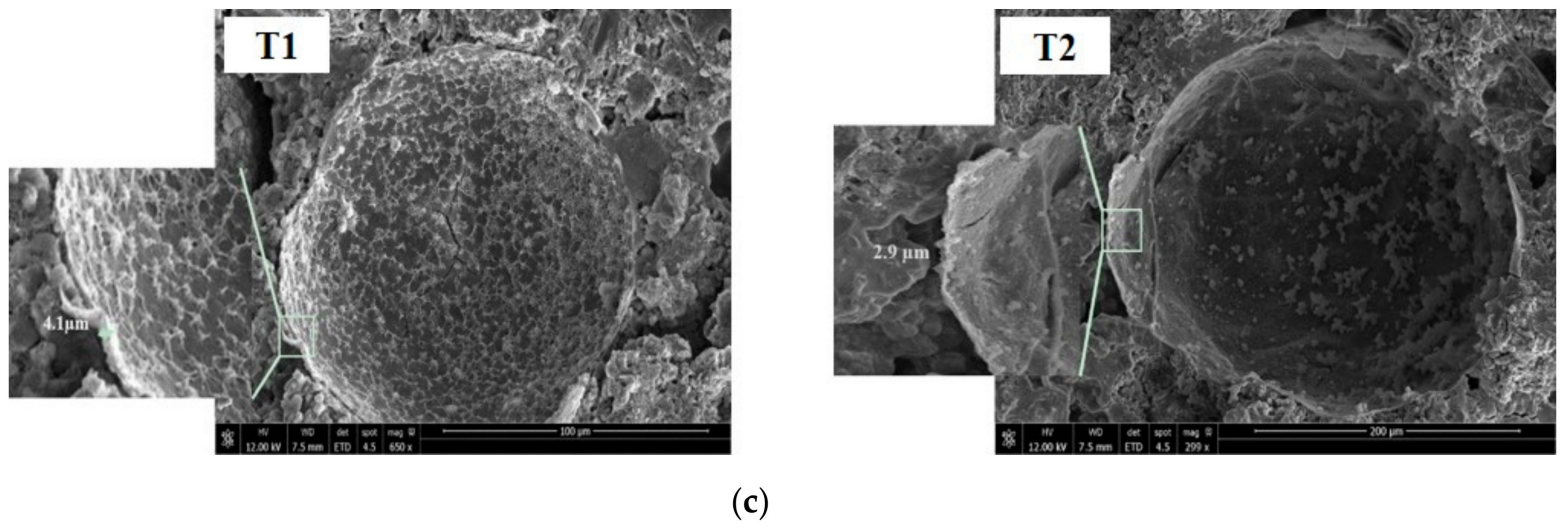
Survivability of the T1 microcapsules in the cement paste [41] (**a**) the microcapsules on a glass slide, (**b**) the microcapsules under the microscope and (**c**) showing good bonding at the interface of the microcapsule with the cementitious matrix.

A number of images are shown in Figure 9 [41] related to the survivability of the T1 microcapsules, those with the stiffer shell, when mixed with the cement. As shown in Figure 9a, the microcapsules mixed into the cement slurry could be observed on the glass slide showing that the microcapsules were sufficiently robust to survive the aggressive mixing process. Under the SEM, Figure 9b, the remaining hemispheric shells of ruptured microcapsules can be seen to be uniformly distributed throughout the cement matrix, which further confirmed their survival during the mixing process. A clearer view of the microcapsules is shown in Figure 9c demonstrating the formation of good bonding at the interface between the microcapsules and the surrounding cementitious matrix and showed good fracture behavior when triggered for healing.

### 3.4. Effect of Microcapsules on Compressive Strength of the Oil Well Cement

**Figure 10:**
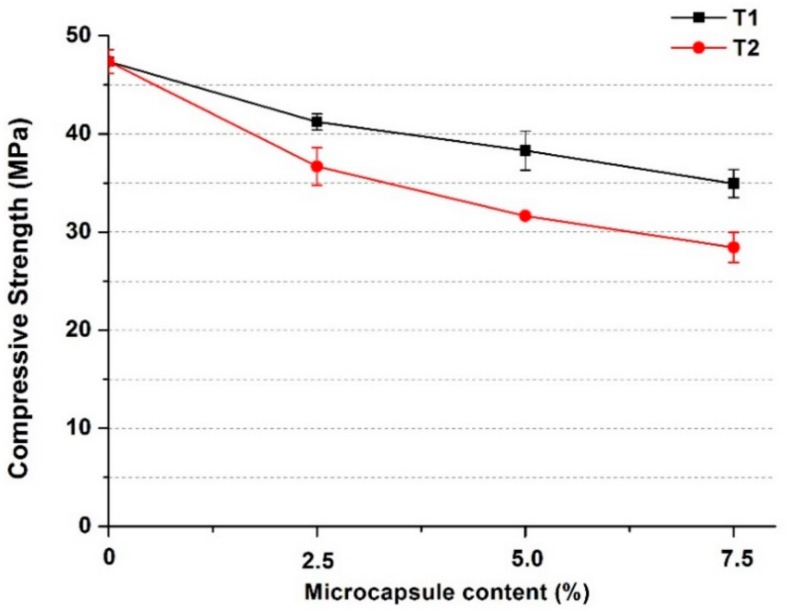
The compressive strength of oil well cement pastes containing a varying content, 0–7.5% by weight, of the T1 or T2 microcapsules after 3 days curing at 80 °C.

As shown in Figure 10, for both types of microcapsules, their addition generally caused a decrease in the compressive strength of the oil well cement pastes, which became more significant as the microcapsule content increased. When the dosage increased to 7.5%, the compressive strength of the cement pastes with T1 decreased by ~26%, while the strength reduction with T2 was higher at ~40%. The small margin of error observed is also an indirect indication of the uniform distribution of the microcapsules within the cement matrix. The decrease in compressive strength is mainly due to the much lower stiffness of the microcapsules compared to the cement matrix [42,43]. Hence the microcapsules formed weaker spots becoming defects in the cement matrix, leading to a decrease in strength. The higher decrease in strength of the T2 microcapsules over the T1 microcapsules is also consistent with their relative stiffness, namely T1 being much stiffer than the T2 microcapsules. Based on the above tests, the T1 microcapsules with the rigid shells were considered more favorable for use in the oil well cement system. Therefore, prismatic cement samples containing the T1 microcapsules were cast for further tests in terms of their self-healing performance 80 °C.

### 3.5. Self-Healing Efficiency of Microcapsules in the Oil Well Cement System

#### 3.5.1. Crack Depth Measurements

**Figure 11:**
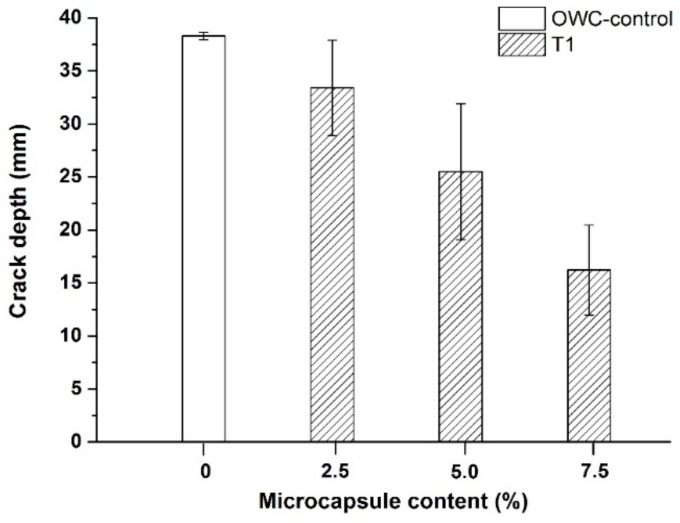
The ultrasonic crack depth measurements of cracked oil well cement prisms containing varying dosages of the T1 microcapsules, from 0 to 7.5% by weight of cement, after healing for 7 days at 80 °C.

The results of the measured crack depths of the oil well cement samples after healing for 7 days at 80 °C are shown in Figure 11. All the cement samples containing microcapsules showed lower crack depths than the control samples after healing. Increasing the content of the microcapsules achieved greater reduction in the crack depth. Increasing the addition of the T1 microcapsules from 2.5% to 7.5% resulted in increased crack depth reduction from ~13% to ~58%. The increased crack depth reduction suggests that there was improved healing inside the cracks.

Mostavi et al. [19] also observed a crack depth reduction of 35% after 14 days of healing achieved by adding 5% (by weight of cement) of sodium silicate microcapsules in cement mortar samples. Giannaros et al. [16] reported that increasing the proportions of sodium silicate microcapsules up to 10.7% (by weight of the cement) resulted in crack depth reduction of 68% after 28 days of healing of mortar prisms. At similar dosages of sodium silicate microcapsules, the oil well cement samples here cured at the higher temperature achieved comparable levels of crack depth reduction as those samples cured at ambient temperature in the aforementioned studies but after a shorter healing period of 7 days. This indicates that the higher temperature curing may have induced fast healing. Although high temperature curing means that very little autogenous healing of cement samples would take place through further hydration, it can however produce accelerated autonomic healing processes based on the reaction of sodium silicate agents with the cement hydration products. In the literature cases referenced, the healing process involved contributions both from autogenous self-healing, by continued cement hydration and precipitation of calcium carbonates for curing underwater, and from autonomic self-healing by the reactions of the sodium silicate with the hydrated cement. By contrast, in the case of oil well cement samples cured at 80 °C, the healing process largely depended on the autonomic healing by the sodium silicate microcapsule reactions while the contributions from autogenous healing were very limited. Considering the challenging conditions for self-healing at high temperatures, the crack width healing achieved by adding the T1 microcapsules can be still seen as a significant improvement compared to the control samples.

#### 3.5.2. Recovery in Water Tightness

**Figure 12:**
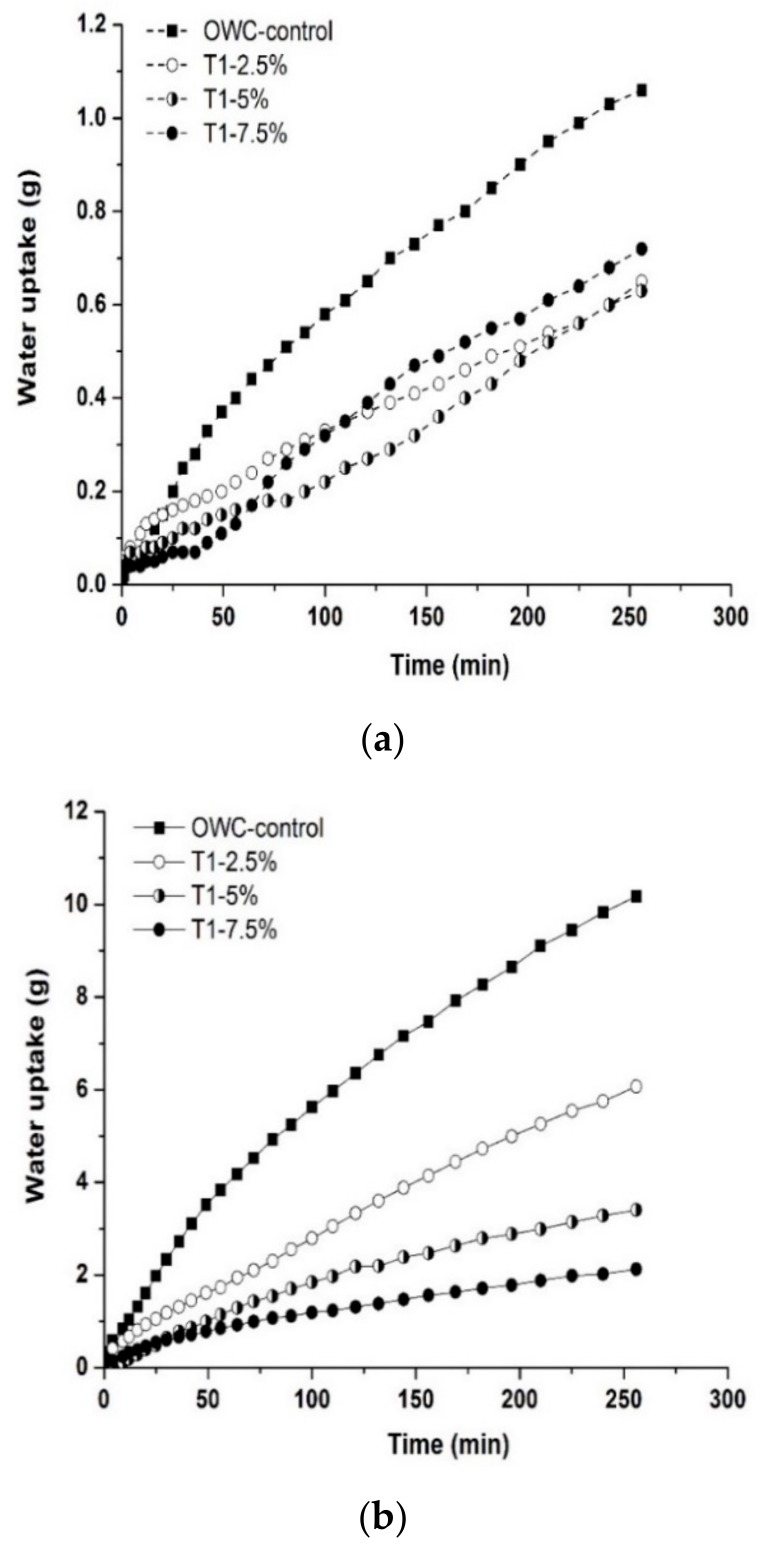

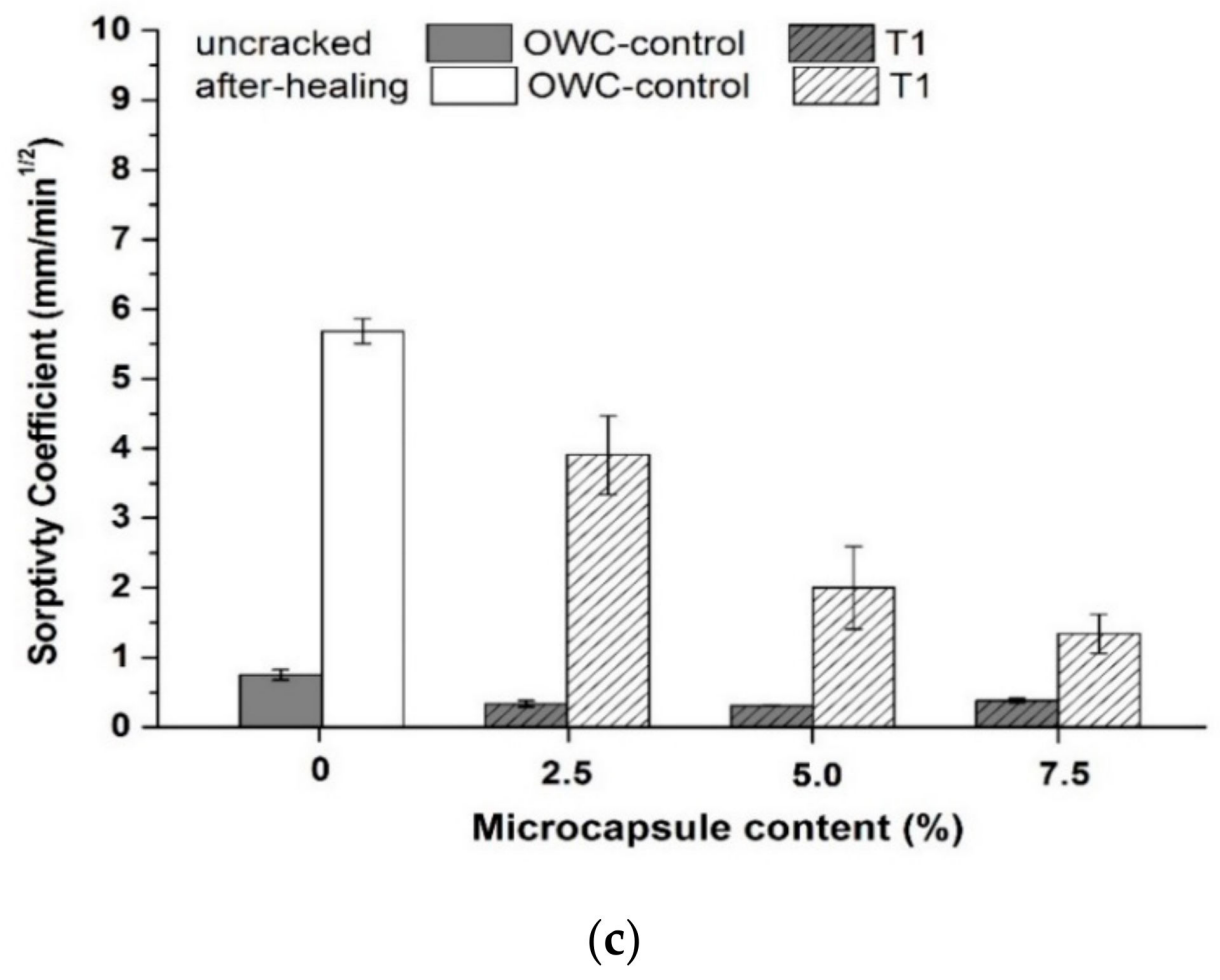
Sorptivity test results (**a**,**b**) the capillary sorptivity in terms of water uptake of the oil well cement prisms with varying dosage of the T1 microcapsules after healing for 7 days at 80 °C (**a**) for the cracked samples and (**b**) uncracked samples and (**c**) the corresponding sorptivity coefficients.

Figure 12 presents the water sorptivity results of the oil well cement prism samples with the T1 microcapsules after healing for 7 days at 80 °C. Figure 12a shows that the water uptake by capillary sorption through the cracks was substantially reduced in the post-healing cement samples containing microcapsules, compared to the control samples, which indicates a recovery in water tightness after healing. The sorptivity coefficients of post-healing cement samples with microcapsules were consistently lower than the control samples, as shown in Figure 12c. At a dosage of 2.5%, the sorptivity coefficients of post-healing T1 cement samples reduced by ~31% compared to the control samples. As the microcapsule dosage increased to 7.5%, the reduction in the sorptivity coefficients reached ~76%. This decreasing trend was not linear, as reduced reduction in sorptivity coefficients was gained by increasing the microcapsule dosage from 5% to 7.5%. This is possibly due to the large volume of voids occupied by the ruptured microcapsules after healing that could create connecting pores within the cracking area of the matrix, and which may increase the sorptivity.

In addition, the hydrophobic polyurea microcapsule shell material appeared to reduce the sorptivity to some extent, as indicated by the water uptake measurements (Figure 12b) as well as the sorptivity coefficients of the uncracked samples (Figure 12c). The characteristics of reduced water absorbency of the cement matrix can be beneficial to its durability in water tightness.

### 3.6. Recovery in Flexural Strength

**Figure 13:**
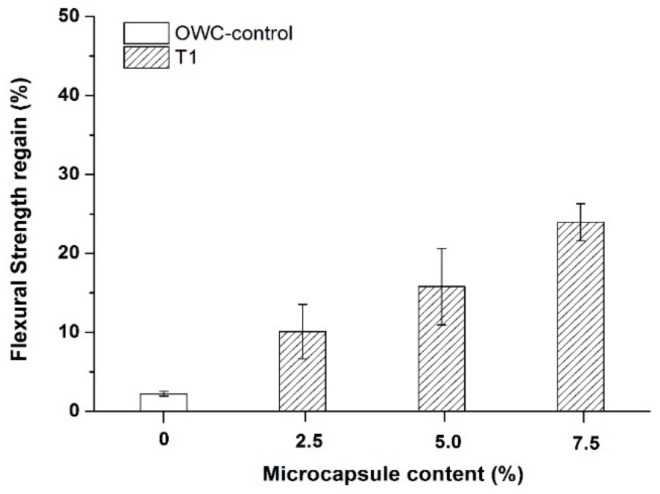
Flexural strength recovery of the cracked oil well cement prisms containing varying dosages of the T1 microcapsules after healing for 7 days at 80 °C.

After use for the capillary sorptivity measurements, the post-healing prism samples were re-loaded under three point bending to examine their flexural strength regain. As shown in Figure 13, the addition of the T1 microcapsules increased the regain in the flexural strength of the oil well cement samples after healing for 7 days at 80 °C. The control samples obtained a flexural strength regain less than ~3%. Such low strength recovery is not surprising since the contributions from autogenous healing is very limited at the high curing temperature. The addition of the T1 microcapsules from a dosage of 2.5% up to 7.5% improved the strength regain by ~7% to 27%. The higher microcapsule content resulted in a greater strength regain. In the autonomic healing by sodium silicate, the main healing product, namely C-S-H, is similar to the main hydration phase of cement which is considered to be the main strength giving component of the hardened cement matrix. The formed C-S-H can provide effective binding within the cracking area, thus contributing to the strength recovery.

### 3.7. Microstructural Analysis

**Figure 14:**
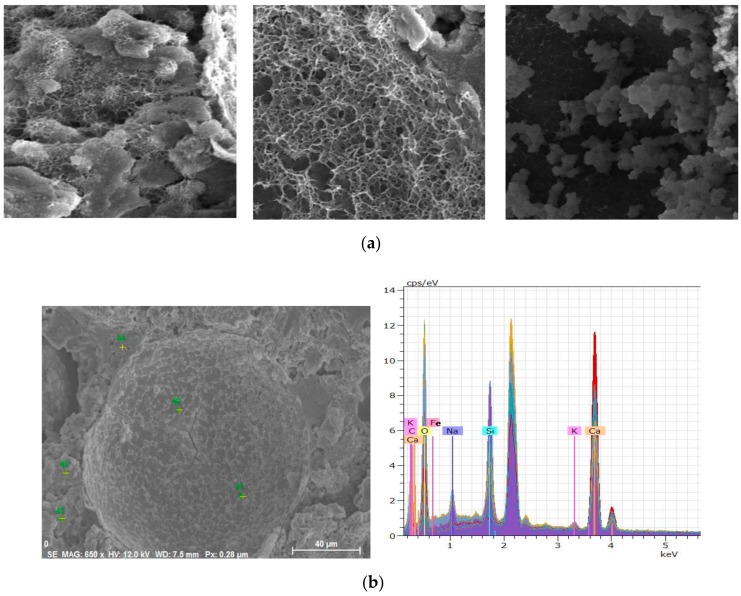
SEM observations of the healed oil well cement pastes containing T1 microcapsules (**a**) various SEM images from the fractured surfaces and (**b**) an SEM image of a microcapsule and the corresponding EDX analysis results of ruptured microcapsules.

SEM images following the healing period are shown in Figure 14a. A clusters of fibrous products were observed around the residual shells of the T1 microcapsules on the cracking surfaces which was found to be C-S-H. Within the shells, there were also small quantities of C-S-H-like products attached to the internal shell wall. To further analyze these healing products, energy-dispersive X-ray spectroscopy (EDX) was used to determine their elemental composition. EDX analysis was carried out on different locations both inside, outside, and around the ruptured microcapsules. As shown in Figure 14b, a range of chemical elements, mainly calcium, silicon, oxygen, and sodium, were detected in those fibrous products inside or around the microcapsules. Those fibrous products were therefore verified as C-S-H which contained calcium, silicon, and oxygen as the main chemical elements. Sodium was a unique element from the sodium silicate core materials. Its presence in the C-S-H products around the microcapsules further confirmed the successful release of sodium silicate to the cracking surface for the healing reaction with calcium hydroxides. Additionally, traces of carbon also indicated the formation of calcite as healing products.

## 4. Conclusions

This paper presented details on the development and application of polyurea microcapsules, containing sodium silicate as cargo, for self-healing in oil well cements at 80 °C. The conclusions from this work are:

1. The two types T1 and T2 of the polyurea microcapsules showed good thermal stability up to ~150 °C and good resistance to high alkalinity in combination. The characterisation results verified their suitability for use in oil well cements under high temperature wellbore conditions at 80 °C;
2. The addition of the T1 and T2 microcapsules led to increase in the plastic viscosity and yield stress of the oil well cement slurries, particularly at dosages above 5% by weight of cement.
3. The compressive strength decreased for increasing additions of both microcapsules. The main difference was in the extent of the decrease in the compressive strength of the oil well cement pastes. he addition of the T2 microcapsules with their rubbery shells led to greater strength reduction than the T1 microcapsules with their rigid shells. The T1 microcapsules were therefore considered more favorable for use;
4. Based on the measurements of crack depth and water sorptivity, it was evident that the addition of the T1 microcapsules significantly improved the self-healing efficiency of oil well cement at 80 °C compared to the reference samples. The healing efficiency generally increased with microcapsule content as more healing agents were provided for healing. The addition of microcapsules at a content of 7.5% achieved crack depth reduction up to ~58%, and the reduction in sorptivity coefficient reached ~76%;
5. The sodium silicate microcapsules also showed effectiveness in improving strength recovery. The C-S-H gels formed act as effective bindings within the cracking area, thus contributing to the strength recovery. The addition of microcapsules (T1 or T2) improved the strength recovery by ~27% at a content of 7.5%;
6. When cured at high temperature of 80 °C, the healing process largely depended on the autonomic healing by the sodium silicate microcapsule reactions while the contributions from autogenous healing were very limited. The higher temperature curing may have also induced fast healing;
7. The microstructure analysis through SEM observations confirmed the survivability of microcapsules in oil well cement system and the successful release of the sodium silicate healing agent. The formation of main healing products C-S-H was also verified by the EDX analysis.

These results provided insights for the first time on the promising application of sodium silicate cargo polyurea microcapsules in the development of self-healing oil well cement for downhole applications at elevated temperatures of 80 °C.
